# Prevalence, Distribution, and Diversity of *Salmonella* Strains Isolated From a Subtropical Lake

**DOI:** 10.3389/fmicb.2020.521146

**Published:** 2020-09-10

**Authors:** Osiris Díaz-Torres, Ofelia Yadira Lugo-Melchor, José de Anda, Misael Sebastián Gradilla-Hernández, Bianca A. Amézquita-López, Demetrio Meza-Rodríguez

**Affiliations:** ^1^Unidad de Servicios Analíticos y Metrológicos, Centro de Investigación y Asistencia en Tecnología y Diseño del Estado de Jalisco, Guadalajara, Mexico; ^2^Departamento de Tecnología Ambiental, Centro de Investigación y Asistencia en Tecnología y Diseño del Estado de Jalisco, Guadalajara, Mexico; ^3^Tecnologico de Monterrey, Escuela de Ingenieria y Ciencias, Zapopan, Mexico; ^4^Facultad de Ciencias Químico Biológicas, Universidad Autónoma de Sinaloa, Culiacán Rosales, Mexico; ^5^Departamento de Ecología y Recursos Naturales, Universidad de Guadalajara, Autlán de Navarro, Mexico

**Keywords:** *Salmonella* distribution, *Salmonella* serotypes, antimicrobial resistance, pulsed-field gel electrophoresis, lake

## Abstract

This study investigated the prevalence, serovar distribution, antimicrobial resistance, and pulsed field gel electrophoresis (PFGE) typing of *Salmonella enterica* isolated from Lake Zapotlán, Jalisco, Mexico. Additionally, the association of the presence of *Salmonella* with physicochemical and environmental parameters was analyzed using Pearson correlation analysis and principal component analysis (PCA). *Salmonella* spp. were identified in 19 of 63 (30.15%) samples. The prevalence of *Salmonella* was positively correlated with air temperature, electrical conductivity, pH, and dissolved oxygen and negatively correlated with relative humidity, water temperature, turbidity, and precipitation. The predominant serotype identified was Agona (68.48%), followed by Weltevreden (5.26%), Typhimurium (5.26%), and serogroup B (21.05%). Overall, the highest detected antimicrobial resistance was toward colistin (73.68%), followed by sulfamethoxazole (63.15%), tetracycline (57.89%), nalidixic acid (52.63%), and trimethoprim (52.63%). All *Salmonella* strains were genetically diverse, with a total of 11 *Xba*I and four *Bln*I profiles on PFGE. The use of these two enzymes allowed differentiate strains of *Salmonella* of the same serotype. The results obtained in this study contribute to a better understanding of the *Salmonella* spp. ecology in an endorheic subtropical lake and provide information for decision makers to propose and implement effective strategies to control point and non-point sources of pathogen contamination.

## Introduction

Among the important lakes in Mexico, Lake Zapotlán was recognized by the Convention on Wetlands as the 1466 Ramsar site in 2005. Hence, this lake acquired a status of national action and international cooperation for the conservation and rational use of its wetlands and resources, to recognize its significant value not only for the country, but for humanity (RAMSAR, 2007). This category was awarded because the lake basin is rich in plant and animal species (Comisión Nacional de Áreas Naturales Protegidas [CONANP], 2005). The lake also hosts important migratory waterfowl in the wetland areas that surround the lake (Shear and de Anda, 2009). Furthermore, it represents an important regional water resource for agricultural purposes, small scale fish harvesting, aquatic sports, and other recreational activities (Secretaría de Medio Ambiente y Desarrollo Territorial, 2005, 2013).

The main sources of water pollution in Lake Zapotlán are the scant treatment of municipal sewages discharged by wastewater treatment plants located in the municipalities of Ciudad Guzman (≈225 L/s) and Gomez Farias (≈30 L/s; Comisión Estatal del Agua [CEA], 2015a,b). Additionally, organic and nutrient contamination reaches the lake from agricultural fields and cattle farms settled around the lake, as well as the eroded material dragged down due to accelerated basin deforestation (Ortíz-Jiménez et al., 2005, 2006). In recent years, several national and international companies have dramatically changed the landscape, with extensive deforestation for the industrial use of wood and to introduce avocado and berries plantations (García de Alba-García, 2006). Moreover, rainwater runoff from the entire basin carries other waste from clandestine or poorly regulated urban landfills have contributed to the lake water quality degradation (Diputados Ciudadanos, 2016). Lake Zapotlán presents endorheic features; in other words, it is in an area of land in which the water that falls or runs through that place has no outlet to another river basin, nor to the sea, nor by infiltration intro layers of groundwater (Comisión Nacional de Áreas Naturales Protegidas [CONANP], 2005; Secretaría de Medio Ambiente y Desarrollo Territorial, 2005). Due to these features, the sources of pollution of this lake severely affect the quality of water used for productive and recreational purposes, a factor that increases the risk for human disease through the consumption of contaminated food or thorough contact with the water source (Greenberg et al., 2008; Malczyk and Branfireun, 2015).

*Salmonella enterica* represent zoonotic bacteria that are distributed throughout the world; they include many serotypes with different host specificity and the apparent ability to cause disease in those hosts is also serovar dependent (Andino and Hanning, 2015). *S. enterica* is one of the main causes of foodborne diseases, causing gastrointestinal diseases, and is also the etiological agent of more serious systematic diseases such as typhoid and paratyphoid fever (Gal-Mor et al., 2014; Pond and World Health Organization [WHO], 2005). In Mexico, there has been high morbidity associated with salmonellosis, with a total of 129,002 cases in 2012 (Dirección General de Epidemiología [DGEPI], 2012). In Jalisco State, there has been an increase in the number of reported salmonellosis cases, with 76,855 in 2016 and 91,173 in 2017 (Dirección General de Epidemiología [DGEPI], 2016, 2017).

*Salmonella* is present in marine and fresh environmental surface waters (Heinitz et al., 2000; Crim et al., 2014), but acute contamination of these environments may come from one or multiple routes, such as wastewater treatment plant discharges, urban and/or agricultural runoff pollution, overburdened septic systems, or contact with local and migratory fauna (Borchardt et al., 2003; Brands et al., 2005; Callaway et al., 2005). Due to the persistence of *Salmonella* in environmental waters, there might be a greater public health concern than previously thought. In some cases, growth outside host organisms leading to the probability of survival between hosts (Cherry et al., 1972; Winfield and Groisman, 2003). In line with *Salmonella* water pollution, surface waters used for irrigation have been identified as a source of contamination of horticultural crops related to outbreaks, including some in Mexico (Centers for Disease Control and Prevention [CDC], 2008, 2013, 2019). Raw or minimally processed vegetables may be contaminated with *Salmonella*, with subsequent direct infection of consumers or cross-contamination of other foodstuffs; which could represent a severe health risk (Quiroz-Santiago et al., 2009).

There is a wide variety of stressors to which *Salmonella* shows a high degree of resistance. Hence, they have a great capacity to persist in changing environments (Winfield and Groisman, 2003), including solar radiation, nutrient starvation, temperature variations, and pH changes, among others. All of these factors influence the survival, persistence, and distribution of these pathogenic bacteria (Xu et al., 1982; Tholozan et al., 1999; Gupte et al., 2003; Cook and Bolster, 2007; McEgan et al., 2013). Rainfall is positively correlated with cases of *Salmonella* infection in subtropical and tropical regions (Zhang et al., 2010). Particularly intense rainfall events may affect the prevalence and degree of contamination by these bacteria, increasing the transport of *Salmonella* from point and non-point sources to superficial water sources (Simental and Martinez-Urtaza, 2008; Zhang et al., 2010). However, relationships between the presence of *Salmonella* in the environment with physicochemical and environmental factors are not yet fully understood.

Phenotypic and genotypic methods are required to determine the origin of the contamination of pathogenic microorganisms in a specific habitat; these techniques allow the identification and characterization of microorganism (Scott et al., 2002). Phenotypic techniques for bacterial characterization include serotyping, biochemical profiling, bacteriophage typing, presence of antigens, and antimicrobial susceptibility profiles (Scott et al., 2002). The appearance of *Salmonella* serotypes with antimicrobial resistance, especially against drugs that represent the first line of treatment (ampicillin, amoxicillin, chloramphenicol, and trimethoprim/sulfamethoxazole) has become a serious health hazard (Centro Nacional de Excelencia Tecnológica en Salud, 2012; Chen et al., 2013). In Mexico, there has been evidence of the problem since the 1970s (Olarte and Galindo, 1973; González-Cortés et al., 1974; Zaidi et al., 2014). The detection of multiple drug resistance (MDR) in *Salmonella* isolated from water sources is of great relevance: MDR facilitates the transmission of antibiotic-resistant *Salmonella* to humans (Li et al., 2014). In Mexico, the increase in antimicrobial resistance in *Salmonella* strains obtained from clinical samples, domestic animals, and food has been linked to the indiscriminate use of antibiotics (Miranda et al., 2009; Amábile-Cuevas, 2010; Jiménez et al., 2011). However, the emergence of MDR *Salmonella* strains obtained from natural water sources requires a better characterization.

Pulsed field gel electrophoresis (PFGE) is a genotyping technique used by scientists to produce a DNA fingerprint for the differentiation and grouping of strains of *Salmonella* spp. or other bacterial isolates; it is also used in surveillance networks for foodborne diseases (Centers for Disease Control and Prevention [CDC], 2004, 2016; Lailler et al., 2002; Swaminathan et al., 2001; Tamada et al., 2001). The analysis can be carried out with one, two, or three restriction enzymes and can be used for reliable molecular typing of the strain(s) under study. In addition, typing methods have been used as an important tool to identify the sources of infection or reservoirs of epidemic outbreaks, to understand the clonality among strains and to determine transmission routes (Bou et al., 2011).

This study investigated the prevalence, serovar distribution, antimicrobial resistance, and PFGE typing of *S. enterica* isolated from Lake Zapotlán, Jalisco, Mexico, as well as the association between the prevalence and dissemination of *Salmonella* and different physicochemical and environmental factors. This information may be useful to develop effective strategies to identify sources of water pollution, to prevent contamination of agricultural food products through irrigation, and to reduce the incidence of water borne diseases in analogous freshwater ecosystems.

## Materials and Methods

### Study Site

**FIGURE 1:**
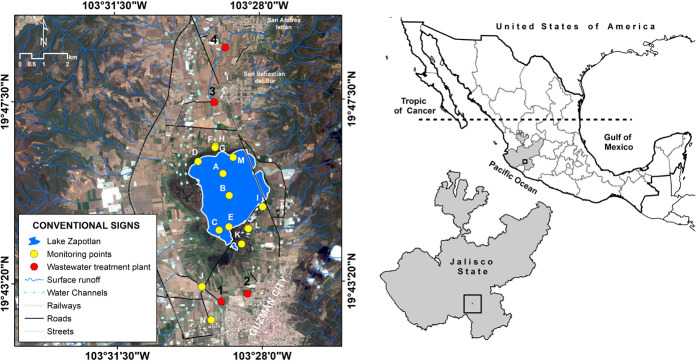
Location of the sampling stations in Lake Zapotlán.

Lake Zapotlán is a small (1,100 ha), perennial, and subtropical water body; it is situated in the western part of Mexico (19°45′N 103°29′W), to the south of the State of Jalisco, between the territorial portion of the municipalities of Gomez Farias and Ciudad Guzman, at 1520 m above sea level (Figure 1). Furthermore, it is classified as an endorheic lake, with a total area of 445 km^2^, a maximum depth of 4.75 m, and an average storage volume of 70.89 Hm^3^ (Comisión Nacional de Áreas Naturales Protegidas [CONANP], 2005; Greenberg, 2009).

### Field Sampling and Monitoring

A total of 63 water samples were collected monthly from June to October 2016 from Lake Zapotlán, from 14 defined sampling stations (A through N) (Figure 1 and Supplementary Table S1). Nine sampling sites (F through N) are located within the main channels that connect the lake to the Gomez Farias and Ciudad Guzman municipalities, which are the main settlements that directly discharge wastewaters into the lake. Furthermore, three sites (C, D, and E) are in the wetlands area and two (A and B) are in the middle of the lake.

### Sampling for Microbiological Analysis

Samples for microbiological analysis were taken from the sampling stations in the middle of the lake and in the wetlands area (A, B, C, D, and E) at 1 m depth. Additional samples were taken at different depths (2, 3, and 4 m), depending on the maximum depth of the lacustrine bed at the sampling site (Supplementary Table S1). Samples were taken at different depths to assess the presence of the *Salmonella* spp. populations through the water column and to infer the potential routes by which it can reach the lake’s biota and humans in contact with the lake’s water.

Samples for microbiological analysis were also taken from the channels that connect the lake to the main settlements (F through N), but these were taken close to the surface (30 cm depth) using an extendible swing sampler (samples could not be taken at different depths at these sites due to the difficult access; Supplementary Table S1). All samples were placed in sterilized bags containing 10 mg sodium thiosulfate tablets, preserved at 4°C in coolers for transportation, and processed in the laboratory in less than 24 h.

### Physicochemical and Environmental Parameters

Physicochemical parameters were measured using an environmental multi-parameter probe (YSI 6600 V2), which includes sensors to measure temperature, pH, dissolved oxygen (DO), turbidity (TUR), and electrical conductivity (CON; all are verified sensors by the United States Environmental Protection Agency’s [EPA’s] Environmental Technology Verification Program; U.S. Environmental Protection Agency, 2003). The calibration protocol for all sensors of the probe were carried out before the use of the probe in field according the manufacturer protocol (YSI, 2009).

Physicochemical parameters were measured only in the sampling stations in the wetlands area and in the middle of the lake (A, B, C, D, and E; Figure 1) at different depths (1, 2, 3, and 4 m; Supplementary Table S1). For the remaining sampling stations (F through N), it was not possible to measure the physicochemical parameters with the multi-parameter probe because it was difficult to access the streams in the channels, which were surrounded by an aquatic plant commonly called tule (*Schoenoplectus acutus*). In addition, August was the only month in which these parameters were not measured in the sampling stations in the wetlands area and in the middle of the lake, because an important surface area of the lake was inaccessible due to the bloom of the aquatic weed commonly called water hyacinth (*c*; Centro Universitario del Sur, 2015).

Samples were taken during the dry and rainy season of the year to analyze the temporal dynamics of *Salmonella* spp. populations and understand the influence of climatological patterns of populations dynamics. Data on rainfall (mm), air temperature (°C), and relative humidity (%) for the sampling period were obtained from the National Meteorological Service weather station (ID # 766560) (TuTiempo.net, 2016) in Ciudad Guzman. This data matrix was used to calculate monthly values for each month of the study (June, July, August, September, and October).

### Isolation of *Salmonella*

A 25-mL aliquot of each collected water sample was placed in 225 mL of pre-enrichment medium (buffered peptone water; BD Difco, United States) and incubated at 37°C for 24 h. This culture medium is used to revitalize microorganisms that might be affected by different storage processes or by environmental conditions, such as drying processes, exposure to ultraviolet (UV) radiation from sunlight, and/or the medium’s pH is very low (Luna, 1991; Hurtado, 2001). Subsequently, to stimulate the growth of this bacteria and inhibit the development of intestinal and coliform bacteria, 1- and 10-mL aliquots of the incubated pre-enrichment medium were transferred to 9 and 100 mL, respectively, Rappaport Vassiliadis R10 (BD Difco) medium and Selenite Cystine broth (BD Difco) and incubated at 37°C for 24 h (Iveson et al., 1964; Trichopoulos et al., 1972; Merck, 1994). To select typical *Salmonella* colonies and to stop the development of other microorganisms, the enrichment broths were streaked onto plates with selective/differential media: xylose lysine deoxycholate agar (XLA; BD Difco) and Hektoen enteric agar (HE; BD Difco) and incubated at 37°C for 24 h (Stanchi, 2007). From the bacterial growth on each plate, 3–4 suspected colony-forming units (CFUs) were selected based on the typical colony morphology—XLA: A typical *Salmonella* colony has a slightly transparent red halo and a black center, a pink-red zone may be seen in the media surrounding the colonies; HE: *Salmonella* produces transparent green or blue-green colonies with or without black centers and appears as almost completely black colonies (Global Foodborne Infections Network Country Databank, 2010; BD, 2013)—and re-isolated onto the agar mentioned above. Finally, those CFUs were transferred to 5 mL trypticase soy broth (TSB; Difco, United States) and incubated for 24 h at 37°C (Standard Method ISO 6579:2002, 2002).

### Molecular Detection of *Salmonella* by Polymerase Chain Reaction (PCR)

*Salmonella* spp. were confirmed by using a previously reported polymerase chain reaction (PCR) protocol (Chiu and Ou, 1996). A 1.5-mL aliquot from the TSB culture was centrifuged at 16,000 *g* for 5 min, the supernatant was decanted, and 1 mL of molecular grade water was added; this step was repeated twice. After removing the supernatant from the previous step, 200 μL molecular grade water was added to resuspend the cell pellet. Finally, the bacterial suspension was placed in a water bath at 100°C for 5 min to cause cell lysis and release the bacterial DNA. Finally, *Salmonella* spp. were confirmed by the amplification of a 284 base pair (bp) fragment of the *invA* gene (Forward: 5′ ACA GTG CTC GTT TAC GAC CTG AAT 3′; Reverse: 3′ AGA CGA CTG GTA CTG ATC GAT AAT 5′; Chiu and Ou, 1996). To confirm the validity of the PCR results, a positive control was included using the bacterial strain *S. enterica* subspecies *enterica* serovar Typhimurium ATCC 14028. *Escherichia coli* ATCC 25922 was used as a negative control.

### Statistical Analysis

A one-way analysis of variance (ANOVA) was performed to determine whether there were significant differences between sampling points and between months during the study period with respect to physicochemical and environmental parameters (Statgraphics, 2009). Furthermore, to explore the correlation between physicochemical and environmental parameters and the presence of *Salmonella* spp., a Pearson correlation matrix was constructed including physicochemical (pH, DO, TUR, and CON), environmental parameters (rainfall [PREC], air temperature [TA], and relative humidity [HUM]) and the prevalence of *Salmonella* spp. (Murdoch and Chow, 1996; Friendly, 2002).

Principal component analysis (PCA) was performed using the data matrix that comprised the recorded data of the physicochemical and environmental parameters and the prevalence of *Salmonella* spp. in order to identify the most significant inter-correlated variables (Sidharth et al., 2017). Subsequently, the first two principal components (PCs) were interpreted with the aid of biplots, which provide a visual representation of the correlation between the physicochemical and environmental parameters with the presence of *Salmonella* spp.

Biplots are a graphical representation of multivariate data (based on the decomposition of the singular value) that simultaneously plot *n* observations and *p* independent variables in two dimensions. The superposition of these two types plots provides additional information about the relationships between the variables and observations. The lengths of the vectors reflect the variance of the corresponding variables and the correlation of two variables is reflected by the angle between the two corresponding vectors (smaller angles represent greater correlations) (Jolliffe, 2002; Everitt and Hothorn, 2011; Zelterman, 2015).

### *Salmonella* Serotyping

Serotyping of positive *Salmonella* strains was performed using a serum agglutination test according to the Kauffman–White scheme. Polyvalent *Salmonella* O and H antisera were used to obtain a presumptive diagnosis and, subsequently, the definitive designation of the antigen was determined by using monovalent antisera (Popoff and Le-Minor, 2001). Serotyping was carried out at the Enteric Bacteriology Laboratory, from the Bacteriology Department of the Institute of Epidemiological Diagnosis and Reference (“InDRE” by its acronym in Spanish) of the Mexican Ministry of Health in Mexico City.

### Antimicrobial Susceptibility

Susceptibility and resistance of all *Salmonella* strains were carried out through the Kirby–Bauer disk diffusion method according to the guidelines of Clinical and Laboratory Standards Institute (Clinical and Laboratory Standards Institute [CLSI], 2005). An 18–24-h culture grown on TSB was used for antimicrobial resistance testing. A sterile cotton swab was used to create a bacterial suspension mixture in 0.85% saline equivalent to a 0.5 McFarland standard (considered equivalent to approximately 1.5 × 10^8^ CFU/mL). The suspension was then vortexed for 2 s, and a fresh sterile swab was later immersed into the suspension. Excess liquid could drain off by touching the side of the test tube. The entire surface of the Müeller–Hinton agar plate was swabbed edge to edge. Each plate was re-streaked two additional times by rotating the plate a quarter turn to ensure an equal distribution of cells to create a uniform bacterial lawn. Eight classes of antimicrobial agents (aminoglycoside, cephalosporin, penicillin, quinolone, tetracycline, beta-lactam, polypeptide, and sulfonamide; Oxoid, United States) were used in the diffusion assays. The specific antimicrobials used were: chloramphenicol 10 μg (C), nalidixic acid 30 μg (NA), ciprofloxacin 5 μg (CIP), tetracycline 10 μg (TE), ceftazidime/clavulanic acid 30 μg (CAZ), trimethoprim 5 μg (TMP), streptomycin 10 μg (S), sulfamethoxazole/trimethoprim 25 μg (SXT), kanamycin 30 μg (K), amikacin 30 μg (AN), ampicillin 10 μg (AM), gentamicin μg 10 (GM), cephalothin 30 μg (CF), amoxicillin/clavulanic acid 20/10 μg (AMC), colistin 25 μg (CL), and imipenem 10 μg (IPM). Using sterile forceps, antimicrobial disks were placed on the agar surface within 5 min of the application of bacterial cells. The plates were further incubated inverted for 16–18 h at 37°C (Clinical and Laboratory Standards Institute [CLSI], 2005).

Following incubation, the lawn was examined to determine whether growth was uniform over the plate. The diameter of the zones of clearing (including the disk) was measured using a Vernier scale. A single colony or a faint haze was regarded as no growth within the zone of clearing. These results were compared to standardized charts for interpretation, as recommended by the manufacturer of the antimicrobial disks and following the criteria of Clinical Laboratory Standards Institute [CLSI] (2005). Results were recorded as susceptible or resistant. Results showing intermediate resistance were considered susceptible in this study, unless otherwise stated. Strains were considered to be MDR if they demonstrated phenotypic resistance to two or more antimicrobial drugs. *E. coli* ATCC 25922 was used as a quality control organism to ensure the validity of the susceptibility testing.

### PFGE

The molecular subtyping of *Salmonella* spp. strains was performed according to the 1-day (24–28 h) standardized laboratory protocol for the molecular subtyping of *Salmonella* by PFGE (Centers for Disease Control and Prevention [CDC], 2017), using *S. enterica* serovar Braenderup H9812 as a standard strain. Briefly, two agarose-embedded DNA samples were digested, each separately, one with a primary enzyme (*Xba*I) and the second with a secondary enzyme (*Bln*I; Roche Diagnostics, Germany) for at least 3 h in an incubator at 37°C. The restriction fragments were separated by electrophoresis in 0.5X Tris-borate-ethylenediaminetetraacetic acid (EDTA; TBE) extended-range buffer (Bio-Rad, United States) with recirculation at 14°C for 20 h and 25 min using a Chef Mapper electrophoresis system (Bio-Rad, United States) with pulse times of 2.20–63.8 s. Following electrophoresis, the gel was stained with GelRed (10,000X; Biotium, United States). Using a gel documenter (Bio-Rad Gel Doc, United States), the DNA bands were visualized under UV light and then photographed and saved as a TIFF image to compare the DNA fragment patterns and cluster analysis, using BioNumerics version 6.6.1.1. software (Applied Maths, Kortrijk, Belgium). Similarity among DNA fragment patterns was determined using the Dice coefficient correlation, and clustering was based on the unweighted pair group method using arithmetic averages (UPGMA) with a band position tolerance and optimization of 1.5%.

## Results

### Physicochemical and Environmental Parameters

Supplementary Tables S2, S3 show the results of the one-way ANOVA for comparing the physicochemical parameters among sampling months (temporal ANOVA) and sampling points (spatial ANOVA), respectively. Supplementary Table S3 shows the results of the one-way ANOVA for comparing the environmental parameters among sampling months.

The results of the temporal ANOVA in Supplementary Table S2 showed that there were statistically significant differences between sampling months for all evaluated physicochemical parameters except pH. With respect to the spatial ANOVA, there were no significant differences for any evaluated parameter (Supplementary Table S3). The results of the one-way ANOVA comparing the environmental parameters among sampling months showed that there were significant temporal variations in HUM and PREC. There were no statistical differences in TA (Supplementary Table S4).

### Presence of *Salmonella* spp.

A total of 19 *Salmonella* strains were confirmed from a total of 63 water samples from all sampling sites: 14 strains were detected (73.68%) in June, three strains (15.78%) in July, and the remaining strains in September (Supplementary Table S5). No strains were detected in August or October. It is important to remark that most of the strains (11/19, 57.89%) were isolated from the samples taken from the surface waters (30 cm; F, G, I, and J), three strains were isolated at 1 m depth, two each at 2 and 4 m, and only one strain was isolated at 3 m (Supplementary Table S6). This finding confirms the facultative anaerobic character of the species in the environment (Abulreesh, 2012).

**FIGURE 2:**
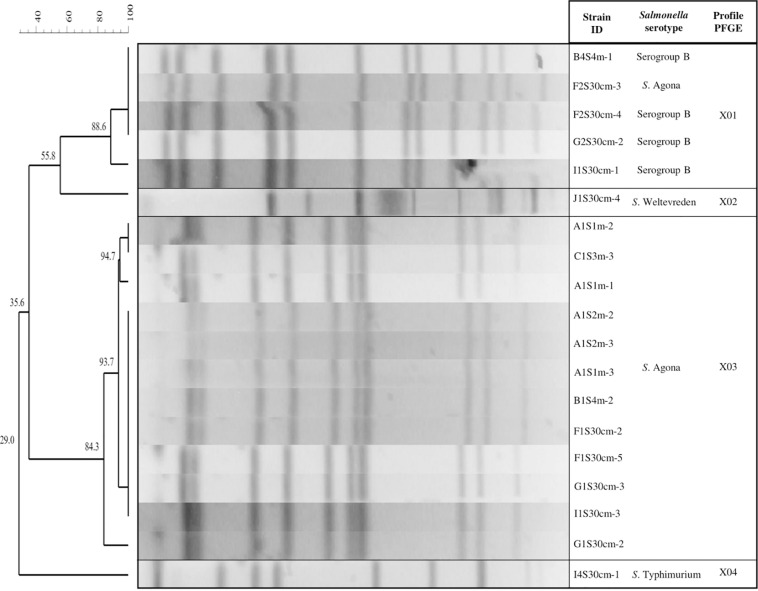
Correlation matrix including the physicochemical and environmental variable values and the presence of *Salmonella*. The correlogram represents the correlation for all pairs of variables. Positive correlations are displayed in blue and negative correlations in red. The intensity of the color is proportional to the correlation coefficient, so the stronger the correlation (i.e., the closer to −1 or 1), the darker the boxes. “*” indicates significant Pearson correlation (*p* < 0.05). The color legend on the right side of the correlogram shows the correlation coefficients and the corresponding colors. WT, water temperature; DO, dissolved oxygen; CON, electrical conductivity; TUR, turbidity; TA, air temperature; HUM, relative humidity; PREC, rainfall, and SAL, *Salmonella*.

The exploratory correlation analysis (Figure 2) suggested that the presence of *Salmonella* (SAL) in Lake Zapotlán was negatively correlated with HUM and PREC while it was positively correlated with TA. With respect to physicochemical parameters, Pearson correlation analysis suggested the presence of *Salmonella* spp. was positively correlated with pH, DO, and CON and negatively correlated with water temperature (WT) and TUR. However, no significant correlations were found between *Salmonella* spp. and the rest of the physicochemical and environmental parameters (*p* > 0.05).

A PCA was also applied to explore the relationship between physicochemical and environmental parameters with the presence of *Salmonella* spp. Four PCs were sufficient to explain the variance of these parameters (Supplementary Table S8). The first four PCs explained 87.37% of the variance (Supplementary Table S7). The highest loadings to PC1 corresponded to DO, HUM, TUR, CON, and TA, all of which are season-dependent variables. The highest loadings of PC2 corresponded WT and PREC, both of which are environmental parameters. PC1 and PC2 accounted for 68.26% of the total data variability. Supplementary Table S8 shows the most influential variables for each of the four components. The graphical analysis of PCA was enhanced by means of biplots, which are the orthogonal projection of the data on the subspace that is spanned by PC1 and PC2 (which contribute the most to the total variance). They describe the importance and correlations between the environmental and physicochemical parameters and the presence of environmental parameters (Gradilla-Hernández et al., 2019). These results reflected the correlation between the presence of *Salmonella* spp. with some of the evaluated physicochemical and environmental parameters.

**FIGURE 3:**
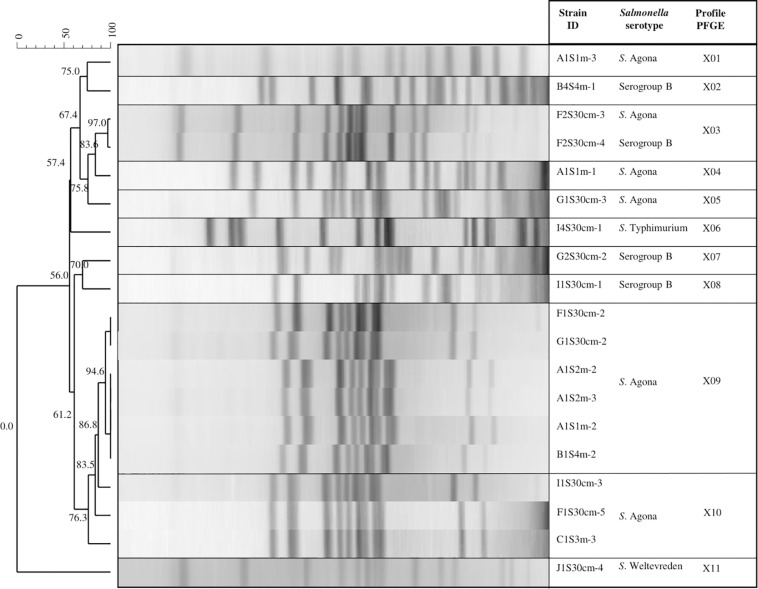
Principal component analysis (PCA) of the physicochemical and environmental parameters; each vector represents a variable, and the correlation of two variables is reflected by the angle between the two corresponding vectors. Abbreviations: WT: water temperature; DO: dissolved oxygen; CON: electrical conductivity; TUR: turbidity; TA: air temperature; HUM: relative humidity; PREC: rainfall; and SAL: *Salmonella*. Code of the isolated *Salmonella* strains: First and/or second letter correspond to the sampling month (Ju: June; Jl: July; S: September; and O: October); the second and/or third letter indicate the sampling site (A, B, C, D, or E); and the penultimate number and letter refer to the depth (1, 2, 3, or 4 m).

On the other hand, the PCA coincided with the results of the correlation analysis. Figure 3 shows how, in a two-dimensional representation composed of PC1 and PC2, the vectors corresponding to the physicochemical and environmental measurements of each sampling month were grouped into clusters. The presence of *Salmonella* spp. was positively correlated with TA, CON, pH, and DO. Conversely, the presence of these bacteria was negatively correlated with the rest of the analyzed physiochemical (TUR and WT) and environmental parameters (PREC and HUM). As mentioned previously, 73.68% of the *Salmonella* strains were isolated in the month of June; Figure 3 presents that temporal groupings (samples per month) influenced season-dependent physicochemical and environmental parameters. These observations suggest that the significant variables in PC1 and PC2 vary among months; these two components sufficiently explain the variability so as to classify the observations among the sampling months.

### Serotyping of *S. enterica*

A total of 19 *Salmonella* spp. strains were isolated and confirmed; only 15 strains were successfully serotyped by the traditional method. All the strains were identified as *S. enterica*. *Salmonella* Agona was the predominant isolated serotype, with 13 of 19 (68.42%) strains; followed by *Salmonella* serogroup B, with 4 of 19 (21.05%) strains; and finally, *Salmonella* Typhimurium and *Salmonella* Weltevreden, each with a single strain.

### Antimicrobial Resistance of Isolated *Salmonella* spp.

The antimicrobial resistance analysis showed that 94.8% (18/19) of the *Salmonella* spp. isolated from the water of the Lake Zapotlán were resistant to at least one antimicrobial agent and 78.9% (14/19) of the strains had MDR to two or more antimicrobial agents. Only 8 of 16 evaluated antimicrobials (colistin, tetracycline, nalidixic acid, sulfamethoxazole, trimethoprim, gentamicin, kanamycin, and streptomycin) generated resistance in *Salmonella* strains. Ten of 19 (52.63%) strains showed resistance to colistin, tetracycline, nalidixic acid, sulfamethoxazole, and trimethoprim, while only 5.26% (1/19) of the strains were susceptible to all the tested antimicrobials. Resistance to colistin was prevalent among the tested *Salmonella* strains (14/19) (Supplementary Figure S1). Our analysis revealed 16 different resistance profiles in the *Salmonella* strains, with colistin present in all resistance profiles (Supplementary Figure S2).

### Genetic Profile of *Salmonella* Serotypes

**FIGURE 4:**
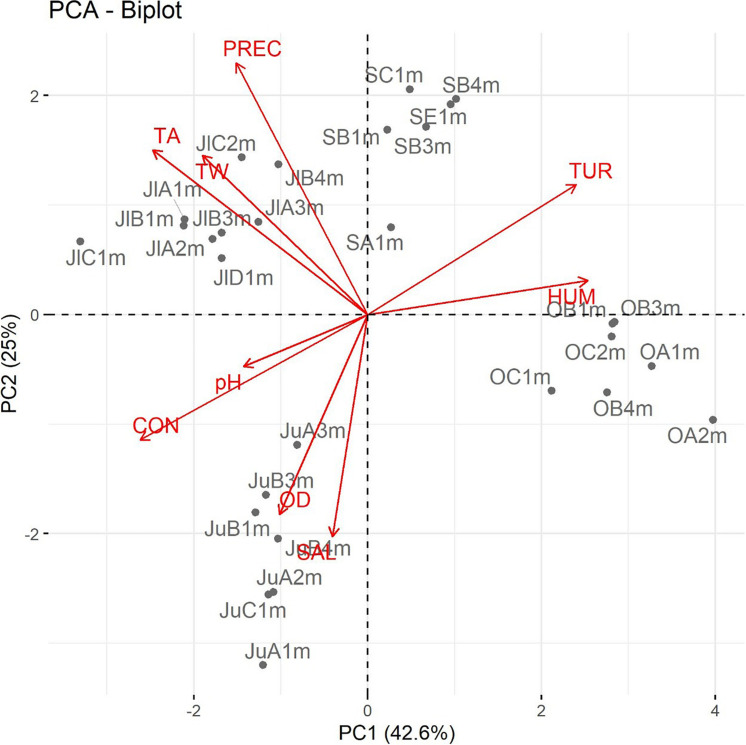
Pulsed field gel electrophoresis (PFGE) *Xba*I dendrogram showing the relationship between *Salmonella* strains. The code for the isolated *Salmonella* strains: The first letter corresponds to the sampling site (A, B, C, F, G, I, or J); the second and third letter indicate the sampling month (1S, 2S, or 4S); the fourth specifies the depth (30 cm, 1, 2, 3, and 4 m); and the dash and number represent an isolated colony within a sample.

The PFGE analysis with the restriction enzyme *Xba*I allowed inter-serotype differentiation to further obtain specific profiles for each. We found that the three serotypes of *Salmonella* (Agona, Typhimurium, and Weltevreden) and the analyzed serogroup B presented 11 genetic profiles (designated X01 to X11; Figure 4). Different genetic profiles were observed for *S*. Agona (five profiles) and serogroup B (three profiles), while the rest (*S*. Typhimurium and *S.* Weltevreden) had a single profile. The X09 profile consisted of five strains isolated from the same sampling date with 94.6% similarity. Three of the strains in this profile (A1S2m-2, A1S2m-3, and A1S1m-2) correspond to the same sampling site, but they were isolated at different depths; the rest were obtained from different sites. In addition, three strains (I1S30cm-3, F1S30cm-5, and C1S3m-3) belonging to the X10 profile were obtained from the same sampling date but at a different sampling points; they had 83.5% similarity. There was 97% similarity in the X03 genetic profile, which consisted of two strains (*S*. Agona and *S.* serogroup B) and corresponded to the same date and sampling point. The results obtained from this serotyping showed that 94.73% of the strains (18/19) belong to *S.* serogroup B, while only one strain belongs to serogroup E (*S*. Weltevreden). However, four of the strains that belong to *S.* serogroup B were not serotyped.

The four isolated *S.* serogroup B strains were distributed in the tributaries that discharge into Lake Zapotlán, while only one strain was isolated from the lake. Profiles X02, X03, X07, and X08 were isolated from different sites and sampling dates. There was no similarity among these profiles: They all had unique band patterns (Figure 4).

**FIGURE 5:**
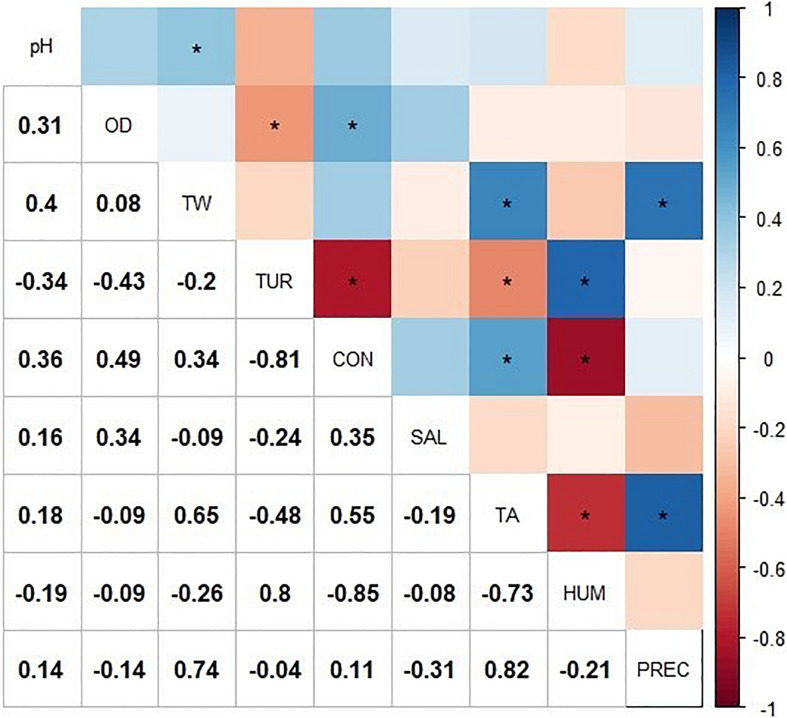
Pulsed field gel electrophoresis (PFGE) *Bln*I Dendrogram showing the relationship between *Salmonella* strains. The code for the isolated *Salmonella* strains: The first letter corresponds to the sampling site (A, B, C, F, G, I, or J); the second and third letters indicate the sampling month (1S, 2S, or 4S); the fourth specifies the depth (30 cm, 1, 2, 3, or 4 m); and the dash and number represent an isolated colony within a sample.

All strains were further characterized using a second restriction enzyme (*Bln*I). The *Bln*I dendrogram shows that all the PFGE patterns were differentiated into groupings of the same serotype or serogroup, except for the X01 genetic profile, which comprises four strains of *S.* serogroup B and one of serotype *S*. Agona; they had 88.6% similarity. These results coincide with the indistinguishable strains of the X03 genetic profile examined with the restriction enzyme *Xba*I that allowed characterization of serogroup B in *S*. Agona strains (Figure 5).

## Discussion

### Presence of *Salmonella* spp.

The percentage of isolates of *Salmonella* spp. obtained in this study were consistent with previously published works, which found a prevalence of approximately 30% when monitoring aquatic environments (Tavechio et al., 2002; Johnson et al., 2003; Arvanitidou et al., 2005). This outcome could be due to the ability of *Salmonella* to persist in nature, particularly when these bacteria are subject to hostile conditions such as resistance to low nutrient availability, drastic fluctuations in temperature and pH, and UV light radiation; even the serotype can determine its survival (Baudart et al., 2000; Steele and Odumeru, 2004; Wilkes et al., 2011; Wanjugi and Harwood, 2012).

The small settlements located near or around Lake Zapotlán discharge raw wastewaters without treatment (Comisión Estatal del Agua [CEA], 2012), which could be a source of *Salmonella* spp., as has been previously demonstrated (Thurston-Enriquez et al., 2002). Other sources of *Salmonella* spp. pollution are manure that result from raising poultry, livestock, pigs, and other domestic animals, all of which are known to be the main reservoirs of *Salmonella* spp. (Morse and Duncan, 1974). During this study, the presence of these animal reservoirs was observed near the sampling sites. Similarly, Gagliardi et al. (2003) analyzed *Salmonella* spp. at different stages of the post-harvest process of cantaloupes and isolated these bacteria from irrigation channels and filters through which the water passed. They suggested a possible environmental or domestic origin of the isolated *Salmonella*. On the contrary, Abulreesh et al. (2004) were unable to isolate *Salmonella* in water samples from a pond that receives direct fecal contamination from waterfowl; however, they were able to detect these bacteria from sediments in the bottom of the pond. This outcome could be attributed, in part, to the concentration of *Salmonella* through sedimentation and also to the greater survival of *Salmonella* in bottom sediments than in water (Fish and Pettibone, 1995; Winfield and Groisman, 2003). These findings possibly suggest that a higher number of *Salmonella* isolates could be found in the sediments of Lake Zapotlán.

On the other hand, the county of Ciudad Guzman has two wastewater treatment plants. These plants are based on activated sludge processes, with capacities of 50 and 150 L/s, respectively; the treated waste is discharged directly into the lake. According to water managers, the treatment plants frequently surpass their capacity during the rainy season because the sewage system of the city is the same used to drive storm waters. Therefore, it is believed that a large amount of wastewater mixed with stormwater reaches the lake with insufficient treatment (Municipal water managers, personal communication, July 05, 2016; Sistema de agua potable de Zapotlán, 2012).

### Relationship Between the Presence of *Salmonella* spp. With Physicochemical and Environmental Parameters

As mentioned in section 2, the physicochemical parameters were neither measured at all the sampling sites nor in August due to poor accessibility because of the presence of aquatic plants in the channels and in the lake. The statistical analysis lacked these physicochemical measurements, which would have strengthened the interpretations regarding the factors that spatiotemporally influence the presence of *Salmonella* in Lake Zapotlán. However, with the measurements obtained from several physicochemical parameters in some sampling sites and through the rest of the sampling period, we can suggest a correlation between these parameters and the presence of *Salmonella* spp. in Lake Zapotlán.

The optimal growth temperature of *Salmonella* spp. have been reported within the range of 35 to 43°C, however, it can multiply in a wider range of temperatures (5.2–46.2°C; International Commission on Microbiological Specifications for Foods, 1996). In this study, although there was more *Salmonella* spp. during the month of June (14/19) (Supplementary Table S5), there were no significant differences in WT parameter during the sampling months (Supplementary Table S2), which could favor the growth of these microorganisms. For instance, the highest average CON was reported for the month of June (Supplementary Table S2); nevertheless, no studies have yet reported a correlation between the presence of *Salmonella* with this variable in aquatic environments. Regarding pH, *Salmonella* spp. has been reported to survive in an environment with a broad pH range (4.05–9.5; Fatica and Schneider, 2011), with an optimal pH of 7–7.5 (Jay et al., 2003). The average pH recorded during the month of June was 8.2 (Supplementary Table S2); however, there were no significant temporal or spatial differences regarding this variable (Supplementary Tables S2, S3). Similarly, McEgan et al. (2013) found that pH was not correlated with the presence of *Salmonella* spp. when comparing results for different sampling sites and sampling months in surface waters across central Florida. In the case of TUR, the lowest average value was registered in June (37.85 NTU; Supplementary Table S2). Other studies have reported that TUR does not affect the prevalence of this bacterium (Morales-Morales et al., 2003; Leskinen et al., 2010; Jimenez and Chaidez, 2011).

The highest DO concentrations are usually present at or near the surface of water bodies (Huang et al., 2019). *Salmonella* spp. is known to be a facultative anaerobic organism for which DO is not a limiting factor for survival (International Commission on Microbiological Specifications for Foods, 1996). In this study, more than 50% of *Salmonella* spp. strains were isolated from samples taken close to the surface (30 cm depth) at the sampling stations located at the main channels discharging into the lake (sampling stations F through N; Supplementary Table S6). This finding suggests that the proximity to the surface (and higher oxygen availability) may be related to the presence of *Salmonella* spp. However, in these sites it was not possible analyze the DO, besides the rest of the physicochemical parameters due to the difficulty in accessing them, therefore, as well as OD, other physicochemical parameters could be better for the survival of this bacterium.

The presence of *Salmonella* spp. in Lake Zapotlán also correlated with environmental parameters (TA, PREC, and HUM). The lowest PREC and HUM were recorded for the months of October and June. As previously mentioned, the greatest prevalence of *Salmonella* (14/19) was observed in June. By contrast, when higher PREC and HUM were recorded for the months of July and September, there was a lower prevalence of this bacterium (5/19; Supplementary Tables S4, S5). These results are consistent with those reported by McEgan et al. (2013): There was a lower concentration of *Salmonella* spp., *E. coli* and fecal coliforms when rainfall was the highest, probably due to the effects of dilution in surface waters. However, it has also been reported that superficial runoff (caused by PREC) may act as an important vehicle for transportation of *Salmonella* spp. to aquatic environments (Levantesi et al., 2012; H. Liu et al., 2018). Finally, when the TA was higher, the highest presence of *Salmonella* was recorded (14/19, June; Supplementary Tables S4, S5). Previous studies have also associated a higher *Salmonella* detection rate in water in warmer seasons (El-Gazzar and Marth, 1992; Polo et al., 1998; Martinez-Urtaza et al., 2004).

### Serotyping of *S. enterica*

In this study, the most predominant serotype was *S*. Agona. This finding may suggest that this serotype had a more efficient adaptation to the aquatic environment in the Lake Zapotlán. In Mexico, the official epidemiological system (Dirección General de Epidemiología) neither reports nor tracks *Salmonella* serotypes that cause gastrointestinal infections in the population (Dirección General de Epidemiología [DGEPI], 2017). However, Gutiérrez-Cogco et al. (2000) reported the most frequent serotypes isolated from human sources in Mexico: *S.* Typhimurium, *Salmonella* Enteritidis, *S.* Agona, and *Salmonella* Typhi were the most frequent; *S*. Agona represented less than 9% of the total of these isolates. In 2004, the WHO through the GFN (World Health Organization Global Foodborne Infections Network, 2010) reported that only 15 strains of *Salmonella* spp. isolated from the environment came from Mexico, from which *S*. Agona was identified five times. Alaniz et al. (1997) highlighted that on farms with cows, pigs, and chickens, the presence of *S. enterica* presented 25 different serotypes. They noted *S*. Typhimurium, in addition to finding these serotypes distributed and shared among these animals. Vásquez et al. (2005) reported that chicken farms are infected by *Salmonella*; the strains isolated from birds were *S*. Typhimurium and *S.* Enteritidis. They concluded that the strains isolated from birds are a source of contamination for humans and a route of dissemination of this bacterium. In another study conducted in China in 2015, the distribution of *Salmonella* serotypes isolated from chickens, pigs, and dairy cows was identified; the most predominant were *S*. Typhimurium, *S*. Enteritidis, and *S*. Agona. The authors mentioned that differences in isolation rates can be interpreted according to the region, sample types, serotypes, collection stations, culture methods, isolation methodologies, culture media, and environmental conditions premises (Kuang et al., 2015).

The diversity of *Salmonella* found in this study can be attributed to several factors, including the ability to survive in a wide range of hosts (Rabsch et al., 2002). Predominant strains of *Salmonella* spp. reflect the adaptability of some serotypes to a specific ecosystem; however, there are serotypes that lack the capacity to survive in the environment for prolonged periods of time. This phenomenon is due to the presence of genetic determinants and to the modification of their physiological characteristics, which gives them the ability to adapt to adverse situations (Winfield and Groisman, 2003). In addition, the ubiquitous nature of these bacteria facilitates their passage from a host to the environment and return to a new host (Winfield and Groisman, 2003). Hence, *Salmonella* is commonly isolated from food, animals, rivers, water, and soil because these reservoirs serve as environments in which bacteria can survive, constituting a permanent source of contamination and facilitating transmission between hosts (Lemarchand and Lebaron, 2002; Winfield and Groisman, 2003).

### Antimicrobial Resistance of *Salmonella* spp.

*Salmonella* strains isolated in this study with MDR indicate the frequent contact in an environment contaminated with antibiotics (Zansky et al., 2002). *Salmonella* and other bacterial groups acquire antimicrobial resistance by random chromosomal mutations, by mutations of existing genes, and through transduction, transformation, and conjugation. These mechanisms involve the transfer of antimicrobial resistance genes from a circulating plasmid, such as R factor, conjugative plasmids, or chromosomal elements (Poppe et al., 1996). Furthermore, the factors that promote resistance to antibiotics in microorganisms can be the indiscriminate use of antibiotics in agriculture, animals, and humans, due to inadequate prescription, lack of following indications of use, and deficiencies in government policies that do not limit the use of antibiotics (Boerlin and Reid-Smith, 2008). Lake Zapotlán, as previously mentioned, represents an important part of fishing, artisanal, agricultural, livestock, and tourism activities (Secretaría de Medio Ambiente y Desarrollo Territorial, 2005). Due to all these activities, the resistance encountered of *Salmonella* spp. to different antimicrobials could be the result of exposure to antibiotics used for human consumption and the control of animals and plants diseases. These diseases could reach the lake through several sources of contamination (point and non-point sources of water pollution; Greenberg et al., 2008; Malczyk and Branfireun, 2015).

In Mexico, antibiotic doses prescribed for therapeutic purposes in humans play a significant role in the increase of resistant strains. Included in this category are self-prescriptions and errors in indications for use, prescriptions when antibiotic use is not justified, and inadequate antibiotic and dose selection, both of which occur when the doctor prescribes antibiotics based only on the patient’s symptoms (Dreser et al., 2008). The frequency of *Salmonella* strains that are resistant to various antibiotics, including ampicillin, tetracycline, streptomycin, sulfamethoxazole/trimethoprim, chloramphenicol, and sulfonamides, as well as other drugs has been widely established in the literature (Levantesi et al., 2012; Abakpa et al., 2014). In particular, high levels of resistance to these antibiotics have been reported in *Salmonella* (Voss-Rech et al., 2016). In Mexico, previous studies aimed at the characterization of antimicrobial resistance have described *Salmonella* strains of clinical (Amábile-Cuevas, 2010) or food (Miranda et al., 2009) origin, or strains that originated in canal waters (López et al., 2009), that are resistant to tetracycline, ampicillin, chloramphenicol, and ceftriaxone. The tetracycline resistance phenotype was also observed in this study: A total of 11 of 19 *Salmonella* strains were resistant to this antimicrobial agent.

Mhongole et al. (2017) determined the antimicrobial resistance profile of *Salmonella* isolated from wastewater and tilapia fish in Tanzania. *Salmonella* serotypes (Kentucky, Chandans, Durban, and Kiambu) were found to be resistant to 8 of 14 tested antimicrobials: sulfamethoxazole (94%), streptomycin (61%), tetracycline (22%), ciprofloxacin and nalidixic acid (17%), trimethoprim (11%), and gentamicin and chloramphenicol (6%). These data coincide with the present study, where 18 strains were resistant to the same antimicrobials. Mhongole et al. (2017) mentioned that wastewater is a source of *Salmonella* contamination in water bodies and food products, including fish and irrigated crops.

Colistin was the only antimicrobial that was present in all resistance profiles (16 profiles) of this study (Supplementary Figure S2). For decades, colistin has been used in veterinary medicine, mainly for the prevention and treatment of enterobacterial infections (Kempf et al., 2016). Morales et al. (2012) analyzed 124 *S*. *enterica* strains from sick pigs; 21% of the isolated strains were resistant to colistin. It has also been widely reported that colistin resistance occurs via point mutation, mostly in genes that affect the lipid bilayer and cell wall biosynthesis (Olaitan et al., 2014). Y.-Y. Liu et al. (2016) first demonstrated a horizontally transferable colistin resistance mechanism: A gene *(mcr-1*) that is resistant to colistin mediated by a plasmid, in strains of *E. coli* and *Klebsiella pneumoniae* originating from different sources (animals, raw meat, and human cases) in China. Doumith et al. (2016) performed a rapid screening of the genomes of *S. enterica* strains isolated from food (two strains isolated from poultry meat) and humans (10 strains isolated from patients). Six *S. enterica* isolates were positive for the *mcr-1* gene from patients who had recently traveled to Asia. We hypothesize that the colistin resistant species found in this study might have their animal and human origin (as previously reported).

### Genetic Profile of *Salmonella* Serotypes

The diversity of *Xba*I PFGE profiles for *S*. Agona and serogroup B might be explained by variations in the genome of the bacterium, because during the process of adaptation to adverse conditions or new environments, the microorganism can integrate new genetic material by the horizontal transfer mechanism (Prosser et al., 2007). Gorski et al. (2011) reported 40 different PFGE profile pulsotypes of *S*. *enterica*, serotypes Give, Typhimurium, Montevideo and Infantis, isolated from different sources (water, wildlife, or soil). These data suggest that they were persistent in that environment. These profiles were found scattered around Monterey County in California, an important agricultural region of the United States.

The PFGE technique has shown limited discriminatory power in subtyping some highly clonal serotypes (e.g., S. enterica serotype Enteritidis and S. enterica serotype Hadar; Swaminathan et al., 2001; Lukinmaa et al., 2004; Hyytiä-Trees et al., 2006). Additionally, PFGE often lacks discriminatory power to partition strains into epidemiologically meaningful clusters. Although this technique is still the current fingerprint method used by PulseNet, they are already transitioning toward using sequencing methods (Centers for Disease Control and Prevention [CDC], 2016), which have a higher discriminatory power than PFGE. In addition, the generated sequencing data is non-ambiguous and portable and can be readily compared between laboratories through the curated website (Hyytiä-Trees et al., 2007). In our study, PFGE was quite cumbersome, subjective, and difficult to perform. Sequencing techniques would have permitted us to avoid some drawbacks presented by the PFGE analysis, such as the strict requirements for DNA preparation, the time invested in performing the technique, and the analysis of the images. However, in this study PFGE showed a high discriminatory power by using a second enzyme (*Bln*I), which allowed us to differentiate PFGE patterns in groups of the same serotype or serogroup. It has been reported that a higher discriminatory power may be achieved by incorporating other potentially more informative enzymes, such as *Bln*I, *Sfi*I, *Pac*I, *Not*I, etc. (Zheng et al., 2007, 2010; Zhao et al., 2008; Son et al., 2013).

The distribution of *S.* Agona and serogroup B can be attributed to the transport and spreading of these bacteria by livestock and wildlife of a locality, through environmental vehicles such as bodies of water (Ferguson et al., 2003). The presence of these animals was observed near the sampling sites during this monitoring; however, it could also be associated to the wastewater that is discharged into the lake. These two sources could have deposited feces of animals and/or infected persons inside the lake and its watercourses, and in this way could have contributed to its contamination. Based on this study, the monitoring of *Salmonella* might facilitate assessments of temporal and spatial factors that are relevant to the incidence of these bacteria, tracking of the source of contamination, higher resolution subtyping, and comparisons with similar data from other agricultural regions in Jalisco. The PFGE analysis provided useful evidence of the clonality of strains *Salmonella* strains from Lake Zapotlán; these data will provide a better understanding of transmission patterns.

## Conclusion

Lake Zapotlán and in the main bodies of water that discharge into it contain diverse *Salmonella* strains. The climatic and water quality features of the lake were correlated with the prevalence and dissemination of *Salmonella* spp. during the study period. The encountered resistance of *Salmonella* spp. to different antimicrobials might be the result of long-term exposure of the bacteria to waters that receive regular loads of antibiotics used for human consumption and control of animal and plants diseases. *Salmonella* diversity and its prevalence in the environment might be related to one or a combination of wastewater effluents, agricultural runoff, and direct fecal contamination from natural fauna. Based on these results, future work must study the main routes of dissemination of this pathogen to explore the factors involved in the presence and dissemination of this bacterium in Lake Zapotlán. The findings of this study can be used to understand *Salmonella* spp. ecology in subtropical regions. This information will aid for proposing and implementing effective strategies to control point and non-point source pollution that is discharged into the lake, as well as to prevent gastrointestinal diseases due to irrigation of food products with contaminated waters.

## Data Availability Statement

The datasets generated for this study are available on request to the corresponding author.

## Author Contributions

ODT, OYLM, and JDA performed the conceptualization and methodology. ODT, MSGH, and DMR performed the software. ODT and JDA performed the validation, supervision, and project administration. ODT and MSGH performed the formal analysis, data curation, and visualization. ODT, OYLM, JDA, and BAAL performed the investigation. ODT prepared and wrote original draft of the manuscript. ODT, OYLM, JDA, and MSGH performed writing and review and editing of the manuscript. All authors contributed to the article and approved the submitted version.

## Conflict of Interest

The authors declare that the research was conducted in the absence of any commercial or financial relationships that could be construed as a potential conflict of interest.
