# Supplementary material for: Prevalence, Distribution, and Diversity of Salmonella Strains Isolated From a Subtropical Lake
Source: Front Microbiol. 2020 Sep 10;11:521146. doi: 10.3389/fmicb.2020.521146 (PMC7518123; doi:10.3389/fmicb.2020.521146)

11. Supplementary Material

Supplementary Table 1. Sampling sites, geographic and municipality location, and sampling depths analyzed in Lake Zapotlán. Sampling place reference symbols—֍: middle of Lake Zapotlán; ₪: wetlands area; ◊: channel.

Sampling site identification	Sampling place reference symbol	Municipality	Geographical coordinates	Depths of microbiological samples	Depths of measurements of physicochemical parameters
A	֍	Ciudad Guzman	19°45'49.0"N 103°28'55.0"W	1, 2, and 3 m	1, 2, and 3 m
B	֍	Ciudad Guzman	19°45'18.67"N 103°28'46.31"W	1, 3, and 4 m	1, 3, and 4 m
C	₪	Ciudad Guzman	19°44'31.27"N 103°29'1.43"W	1 and 3 m	1 and 3 m
D	₪	Gomez Farias	19°46'5.69"N 103°29'30.92"W	1 m	1 m
E	₪	Ciudad Guzman	19°44'35.77"N 103°28'47.11"W	1 and 2 m	1 and 2 m
F	◊	Gomez Farias	19°46'26.83"N 103°29'6.17"W	30 cm	Not measured
G	◊	Gomez Farias	19°46'26.85"N 103°29'5.94"W	30 cm	Not measured
H	◊	Gomez Farias	19°46'24.68"N 103°29'6.17"W	30 cm	Not measured
I	◊	Ciudad Guzman city	19°45'03.0"N 103°27'58.0"W	30 cm	Not measured
J	◊	Ciudad Guzman	19°43'14.0"N 103°29'27.0"W	30 cm	Not measured

K	◊	Ciudad Guzman	19°44'12.0"N 103°28'29.0"W	30 cm	Not measured
L	◊	Ciudad Guzman	19°44'33.0"N 103°28'19.0"W	30 cm	Not measured
M	◊	Gomez Farias	19°46'11.0"N 103°28'40.0"W	30 cm	Not measured
N	◊	Ciudad Guzman	19°42'28.0"N 103°29'14.0"W	30 cm	Not measured

Supplementary Table 2. Physicochemical parameters by sampling month. Abbreviations—WT: water temperature; DD: dissolved oxygen; CON: electrical conductivity; TUR: turbidity; NTU: nephelometric turbidity unit; SD: standard deviation. The analysis of variance (ANOVA) results are presented for comparing the means of physicochemical parameters between months. Red numbers: $P < 0.05$, which is considered statistically significant.

**Month**	**WT (°C)**	**DO (mg/L)**	**CON (mS/cm)**	**TUR (NTU)**	**pH**
June	23.23	6.05	1.02	37.85	8.20
July	23.75	2.72	0.96	40.3	8.07
September	24.08	3.63	0.77	84.05	8.09
October	22.2	2.43	0.72	81.62	7.92
Mean (SD)	23.32 (0.84)	3.64 (1.668)	0.88 (0.12)	58.73 (25.88)	8.07 (0.22)
P	0.00	0.00	0.00	0.00	0.13

Supplementary Table 3. Physicochemical parameters by sampling point measured from Lake Zapotlán sites. Abbreviations—WT: water temperature; DO: dissolved oxygen; CON: electrical conductivity; TUR: Turbidity; SD: standard deviation.

Sampling Point	WT (°C)	OD (mg/L)	CON (mS/cm)	TUR (NTU)	pH
A	22.84	3.72	0.89	49.65	8.05
B	23.48	3.70	0.87	64.18	8.05
C	23.59	3.40	0.87	60.4	8.23
D	23.63	4.43	0.95	30.4	7.72
E	23.89	2.87	0.79	93.5	8.00
Mean (SD)	23.32 (0.89)	3.64 (1.66)	0.88 (0.12)	58.73 (25.88)	8.07 (0.22)
P value	0.43	0.96	0.91	0.33	0.20

Supplementary Table 4. Environmental parameters recorded during the study period. Abbreviations—TA: air temperature; HUM: relative humidity; PREC: rainfall; SD: standard deviation. Red numbers: $P < 0.05$, which is considered statistically significant.

Sampling Month	TA (°C)	HUM (%)	PREC (mm)
June	23.16	56.35	2.98
July	22.3	59.81	7.90
August	22.34	63.76	3.65
September	22.11	63.92	3.86
October	22.32	54.91	0.50
Mean (SD)	22.42 (1.23)	59.98 (10.65)	3.51 (7.49)
P	0.05	0.00	0.04

Supplementary Table 5. Presence of *Salmonella* spp. (number of isolated strains) by sampling month.

Sampling	Month	Presence of *Salmonella* spp. (number of isolated strains)
1	June	14
2	July	3
3	August	Absence
4	September	2
5	October	Absence

Supplementary Table 6. Presence of *Salmonella* spp. at different depths (number of strains isolated).

Depth	Presence of *Salmonella* spp. (number of isolated strains)
30 cm	11
1 m	3
2 m	2
3 m	1
4 m	2

Supplementary Table 7. Principal components (PCs).

Component	Eigenvalue	% of variance	Cumulative % variance
PC1	3.452	38.36	38.36
PC2	2.691	29.90	68.26
PC3	1.046	11.62	79.88
PC4	0.674	7.49	87.37
PC5	0.641	7.12	94.49
PC6	0.259	2.87	97.36
PC7	0.187	2.08	94.44
PC8	0.049	0.54	99.98
PC9	0.001	0.02	100.00

Supplementary Table 8. Principal component analysis (PCA, with four significant principal components [PCs]). Abbreviations—DO: dissolved oxygen; WT: water temperature; TUR: turbidity; CON: electrical conductivity; SAL: *Salmonella*; TA: air temperature; HUM: relative humidity; PREC: rainfall. Bold values indicate the most influential variables for each component.

**Variable**	**PC1**	**PC2**	**PC3**	**PC4**
pH	-0.2775	-0.1690	**0.5086**	**-0.3997**
OD	**-0.3940**	0.1230	0.3432	-0.1966
WT	-0.1217	**-0.5055**	0.3166	0.1668
TUR	**0.4379**	0.1299	**0.3887**	0.1688
CON	**-0.4841**	-0.1499	-0.2630	0.0590
SAL	-0.2921	0.2391	0.1688	**0.8277**
TA	**-0.4577**	0.2844	0.1688	-0.0339
HUM	**0.43235**	0.06908	**-0.44653**	0.04688
PREC	-0.1361	**-0.5160**	**-0.4157**	0.0921

Supplementary Figure 1. Antimicrobial resistance of the isolated *Salmonella* spp. strains.

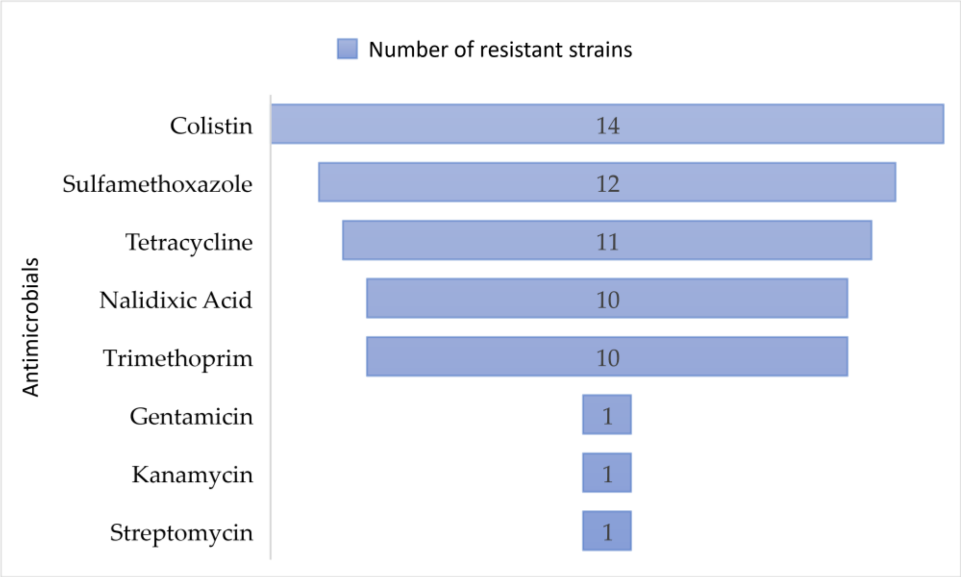

Supplementary Figure 2. Antimicrobial resistance profiles of the isolated *Salmonella* strains. Abbreviations—CL: colistin; TE: tetracycline; TMP: trimethoprim; SXT: sulfamethoxazole; NA; nalidixic acid.

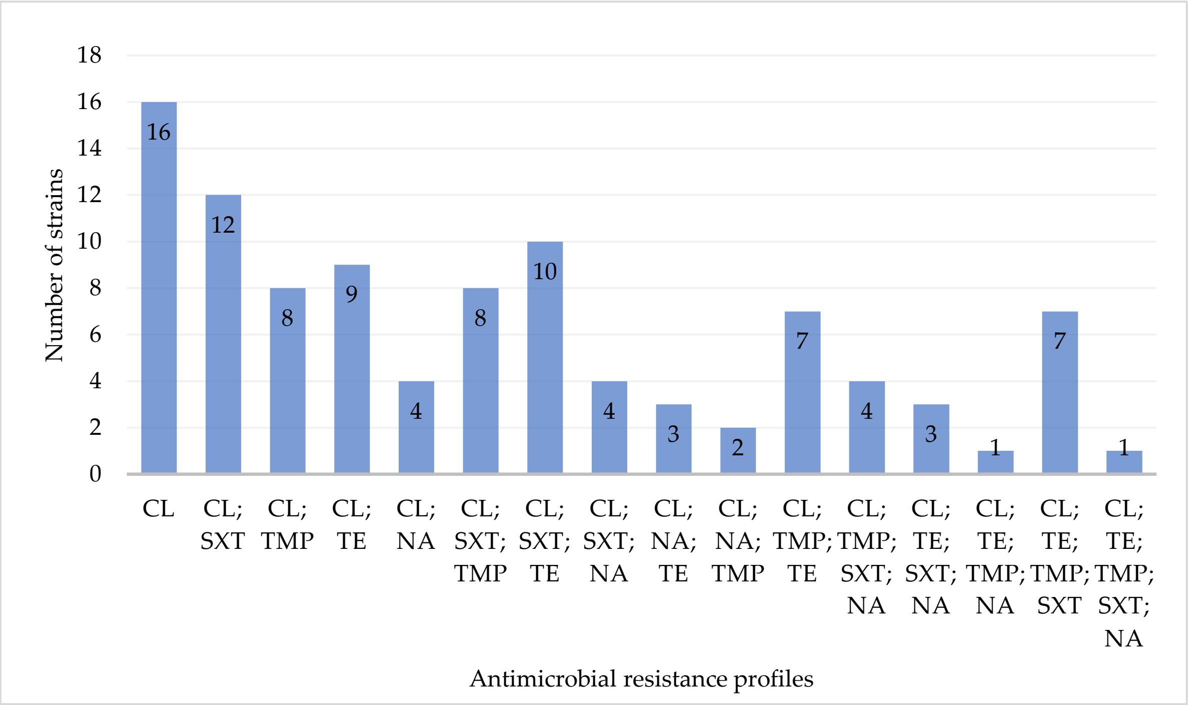

Supplement: Supplementary file 1 [file Data_Sheet_1.PDF]
